# Hydrogen Peroxide Modulates the Timely Activation of Jun and Erk in Schwann Cells at the Injury Site and Is Required for Motor Axon Regeneration

**DOI:** 10.3390/cells14090671

**Published:** 2025-05-03

**Authors:** Samuele Negro, Chiara Baggio, Marika Tonellato, Marco Stazi, Giorgia D’Este, Aram Megighian, Cesare Montecucco, Michela Rigoni

**Affiliations:** 1 Department of Biomedical Sciences, University of Padua, 35131 Padua, Italy; 2 Cancer Neuroscience Laboratory, Francis Crick Institute, London NW1 1ATK, UK; 3 Neurobiology Lab, IRCCS San Camillo Hospital, 30126 Venice, Italy; 4 Padua Neuroscience Center, University of Padua, 35129 Padua, Italy; 5 CNR Institute of Neuroscience, 35131 Padua, Italy; 6 Myology Center (CIR-Myo), University of Padua, 35131 Padua, Italy

**Keywords:** Schwann cells, peripheral nerve regeneration, hydrogen peroxide, ERK1/2, c-Jun

## Abstract

Peripheral nervous system (PNS) neurons, including motor neurons (MNs), possess a remarkable ability to regenerate and reinnervate target muscles following nerve injury. This process is orchestrated by a combination of intrinsic neuronal properties and extrinsic factors, with Schwann cells (SCs) playing a central role. Upon injury, SCs transition into a repair phenotype that allows axonal regeneration through molecular signaling and structural guidance. However, the identity of the SCs’ reprogramming factors is only partially known. We previously identified hydrogen peroxide (H_2_O_2_) as an early and key driver of nerve repair, inducing gene expression rewiring in SCs to support nerve re-growth. In this study, we quantitatively assessed the role of H_2_O_2_ in the activation of key pro-regenerative signaling pathways in SCs following sciatic nerve compression, specifically the extracellular signal-regulated kinase 1/2 (ERK1/2) and c-Jun, which are essential for functional nerve recovery. Notably, we found that H_2_O_2_ neutralization does not impact degeneration, but it significantly affects the regenerative response. Collectively, our findings establish H_2_O_2_ as a promising regulator of the Schwann cell injury response at the injury site, linking oxidative signaling to the molecular mechanisms governing nerve regeneration.

## 1. Introduction

Unlike central neurons, neurons of the peripheral nervous system, including motor neurons (MNs), possess a remarkable ability to regenerate and reinnervate their target muscles [1,2]. Following peripheral nerve injuries, structural and functional repair is governed by a complex interplay of MN intrinsic and extrinsic factors. Initially, neurons must survive the injury and undergo transition from a transmitting state to a growth-promoting state [3,4]. Second, the generation of a cellular permissive environment, with Schwann cells (SCs) at the centre, is crucial for successful regeneration [5,6,7]. Upon nerve injury, both myelinating and non-myelinating SCs convert into a specialized repair phenotype (repair SCs) that: (i) engage phagocytosis to remove nerve debris, (ii) stimulate axonal re-growth, and (iii) physically guide reinnervation and provide molecular signals and spatial cues [6,8]. However, the nature of the precise triggers initiating this response and driving SC differentiation remains largely unknown.

Recent findings from our research identified hydrogen peroxide (H_2_O_2_), produced by neuronal mitochondria, as a key signaling molecule participating in the early response to different types of peripheral nerve injuries. Specifically, we investigated two distinct injury models: in one case, we exploited α-latrotoxin, a neurotoxin that causes a calcium-mediated specific degeneration of motor axon terminals through its pore-forming activity, and another involving sciatic nerve (SN) compression [9,10]. In the latter, we used the cytosolic H_2_O_2_-specific fluorescent probe PF6-AM to monitor real-time H_2_O_2_ production in the sciatic nerves of live, anesthetized mice. We found that H_2_O_2_ levels increased rapidly, within minutes, after nerve crush, near the injury site. Here, we have extended these studies and provided evidence that the rapid H_2_O_2_ production upon SN crush initiates the molecular events driving both functional and structural nerve recovery. Specifically, H_2_O_2_ triggers the early ERK 1/2 activation and c-Jun upregulation, two crucial events responsible for SC reprogramming into the repair phenotype [11,12]. These findings establish H_2_O_2_ as a key regulator of the early nerve injury response, linking oxidative signaling to the cellular mechanisms underlying nerve regeneration.

## 2. Materials and Methods

### 2.1. Ethical Statment

Ten- to twelve-week-old CD1 mice were used for compound muscle action potential (CMAP) recordings, immunofluorescence (IF), and Western blot (WB) analysis. C57BL/6 mice expressing cytosolic GFP under the proteolipid protein (plp) gene promoter were generously provided by Dr. W.B. Macklin (Aurora, CO, USA), with assistance from Dr. T. Misgeld (Munich, Germany), and were utilized for imaging studies. Mice expressing the Tomato fluorescent protein specifically in choline acetyltransferase (ChAT)-positive neurons were generated by crossing C57BL/6 ChAT-Cre knock-in mice with C57BL/6 Rosa26.tdTomato mice (Jackson Laboratories, Bar Harbor, ME, USA). Wistar rats were used for SC cultures. Mice were housed under a 12 h light/dark cycle at a constant temperature, with ad libitum access to water and standard chow. All surgical procedures were performed under general anesthesia via isoflurane inhalation. Paralysis was limited to one hind limb and did not affect food or water intake. All experimental procedures involving animals adhered to the ARRIVE guidelines and were conducted in compliance with ethical standards. Experiments performed in Italy were approved by the ethical committee and the animal welfare coordinator of the OPBA at the University of Padua. Procedures were carried out under projects authorized by the Italian Ministry of Health, Ufficio VI, Rome (authorization numbers: 521/2018 PR; 439/2019 PR; 146/2024 PR; and D2784.N.PCU), in accordance with national legislation (D.L. n. 26, 14 March 2014) and the European Community Council Directive (2010/63/EU) on the care and use of animals for scientific purposes. Animal handling was performed by specialized personnel and supervised by inspectors from the Veterinary Service of the Local Sanitary Service (ASL 16-Padua), acting as local representatives of the Ministry of Health.

### 2.2. Antibodies and Reagents

Antibodies and fluorescent conjugates with relative dilutions: α-BTx Alexa Fluor 555 (B35451 Thermo Fisher, (Waltham, MA, USA, 1:200); anti-pERK for IF and WB (9101S Cell Signaling, Danvers, MA, USA), 1:200 and 1:1000, respectively); anti-ERK for IF and WB (4695S Cell Signaling Danvers, MA, USA), 1:200 and 1:1000, respectively); anti-c-JUN for immunostaining (ab32137 Abcam, 1:200); anti-c-Jun for WB (9165S Cell Signaling, Danvers, MA, USA, 1:100); anti-S100 (Z0311 Dako, Santa Clara, CA, USA, 1:400), anti-NF (ab4680 Abcam, Cambridge, UK, 1:500), and anti-VAMP1 ([13], 1:200) for IF; and anti-Calnexin (ADI-SADI-SPA-860-F Enzo Life Sciences, Long Island, NY;USA) 1:1000) for WB. Secondary Alexa Fluor-conjugated antibodies (1:200) for IF were from Thermo Fisher (Waltham, MA, USA).

### 2.3. Sciatic Nerve Compression

The SN was exposed under general anesthesia at the level of the sciatic notch while preserving the integrity of the gluteal musculature. For nerve compression (crush injury), hemostatic forceps pre-coated with powdered charcoal were used to mark the site of injury. The nerve was pinched 0.5 cm from its proximal insertion at the hip for 20 s at the third click of the forceps. Following the procedure, the gluteal musculature was repositioned, and the skin was sutured using 6–0 braided silk, non-absorbable sutures (ETHLCON2, Biological Instruments, Besozzo, Italy, 8697). A detailed description of the protocol is reported in [14].

Electrophysiological assessments and IF/WB analyses were performed at designated time points following nerve injury. In specific experiments, prior to nerve compression, catalase (C1345, Sigma Aldrich, St. Louis, MI, USA) was intra-sciatically administered (1 mM in physiological solution, 2 μL injection volume) using a pulled graduated glass micropipette. The micropipette was carefully inserted into the medial region of the sciatic nerve beneath the perineurium to ensure targeted delivery [15,16,17].

### 2.4. Compound Muscle Action Potential Recordings

Compound muscle action potential (CMAP) recordings were performed 5, 15, 21, 28, and 35 days after SN crush. The 21–28-day interval corresponded to approximately 40–60% recovery of neurotransmission, making it a suitable time frame for evaluating the effects of treatment on functional nerve recovery [18]. The 35-day time point corresponded to almost 100% recovery. Under general anesthesia, the SN was surgically exposed at the sciatic notch, and a small piece of parafilm was placed beneath the nerve to provide mechanical support while maintaining moisture with phosphate-buffered saline (PBS). A pair of stimulating needle electrodes (Grass, Middleton, WI, USA) was carefully positioned above the crush site using a mechanical micromanipulator (MM33, FST, Heidelberg, Germany) until they made gentle contact with the nerve. Electromyographic recordings of gastrocnemius muscle activity were obtained using a pair of needle electrodes (Grass, USA), with the recording electrode inserted midway into the muscle and the reference electrode placed in the distal tendon. CMAPs were elicited by supramaximal stimulation of the sciatic nerve at 0.5 Hz (0.4 ms stimulus duration) using a stimulator (S88, Grass, USA) connected to a stimulus isolation unit (SIU5, Grass, USA) in capacitance coupling mode. To ensure supramaximal stimulation (ranging from 5 to15 mV in controls to up to 50 mV in nerve-injured mice), stimulus intensity was gradually increased until the CMAP amplitude reached a plateau. The recorded signals were amplified using an extracellular amplifier (P6, Grass, USA), digitized via a digital A/C interface (National Instruments, Austin, TX, USA), and processed for real-time visualization and offline analysis using specialized software (WinEDR V3.4.6, Strathclyde University; pClamp, Molecular devices, San Jose, CA, USA). Final data analysis was conducted using pClamp 8 software.

### 2.5. Immunofluorescence of Sciatic Nerves and Muscle Tissues

SNs were isolated at various time points from crush, with or without catalase injection. For cryosectioning, samples were fixed in 4% paraformaldehyde (PFA) in phosphate-buffered saline (PBS) for 30 min at room temperature (RT). Fixed nerves were cryoprotected in sucrose (30%) overnight and embedded in an optimal cutting temperature (OCT) compound. Samples were gradually frozen in isopentane cooled with liquid nitrogen vapors and sectioned at 20 μm thickness using a Leica CM1520 cryostat. Cryosections were then quenched in 0.24% ammonium chloride (NH_4_Cl) in PBS for 20 min. Following permeabilization and a 2 h blocking step in a solution containing 15% goat serum, 2% bovine serum albumin (BSA), 0.25% gelatin, 0.20% glycine, and 0.5% Triton X-100 in PBS, sections were incubated with primary antibodies in blocking solution for 72 h at 4 °C. After washings, sections were incubated with secondary antibodies for 2 h and mounted using Dako fluorescence mounting medium (Agilent Technologies, Santa Clara, CA, USA, cat. S3023).

For teased fiber preparations, SNs were fixed in 4% PFA for 1 h, washed 3 times in PBS for 5 min each, and manually separated into individual fibers or small bundles. Fibers were then permeabilized and blocked in a solution containing 1% Triton X-100 and 10% BSA in PBS for 1 h at RT. Following blocking, fibers were incubated with primary antibodies in blocking solution for approximately 3 days at 4 °C under mild agitation. After 3 PBS washes at RT, fibers were incubated for ~2 h at RT with the following secondary antibodies: anti-mouse-555 (A-21422 Thermo Fisher Scientific, Waltham, MA, USA. 1:500) and anti-rabbit-647 (A31573 Thermo Fisher Scientific, 1:500). Following additional PBS washes, fibers were mounted using Dako fluorescence mounting medium. Whole-mount nerve staining was performed in selected experiments following previously described protocols.

Soleus muscles were dissected at various time points following SN crush, with or without catalase treatment. Muscles were fixed in 4% PFA in PBS for 30 min at RT and subsequently quenched in 0.24% NH_4_Cl in PBS for 20 min. After permeabilization and a 2 h blocking step in the same blocking solution described for SN cryosections, samples were incubated with primary antibodies against VAMP1 and NFs in blocking solution for 72 h at 4 °C. Following PBS washes, muscles were incubated with secondary antibodies and α-bungarotoxin (α-BTx) Alexa Fluor 555 to visualize post-synaptic acetylcholine receptors (AChRs). Muscle fibers were mounted using Dako fluorescence mounting medium. For both sciatic nerve and muscle preparations, slides were air-dried, and z-stack images were acquired using a Zeiss (Jena, Germany) LSM900 Airyscan2 confocal microscope equipped with an EC Plan-Neofluar 40×/1.30 oil objective or a 5× objective. Laser excitation wavelengths, power intensities, and emission filter settings were optimized to minimize spectral bleed-through between fluorophores. Fluorescence signal quantification was performed using FIJI windows (64-bit)software. When applicable, GFP fluorescence (of plp-GFP-expressing SCs) or DAPI staining were used as masks for signal localization analyses.

### 2.6. Primary Cell Cultures and Treatments

Primary SCs were purified from SNs of six P3 Wistar rats as previously described [19,20]. Cells were exposed to 50 µM H_2_O_2_ in Krebs–Ringer Buffer (KRH: HEPES-Na 25 mM at pH 7.4, NaCl 124 mM, KCl 5 mM, MgSO_4_ 1.25 mM, CaCl_2_ 1.25 mM, KH_2_PO_4_ 1.25 mM, and glucose 8 mM) at 37 °C, and then processed for IF or WB.

For IF, samples were treated as previously reported in [21]. Coverslips were mounted using Dako fluorescence mounting medium and examined using a Zeiss LSM900 Airyscan2 confocal microscope equipped with an EC Plan-Neofluar 40×/1.30 oil objective. Fluorescence intensity was measured using Fiji software. All images were captured under non-saturating conditions, with consistent imaging parameters applied across all samples.

### 2.7. Western Blotting

For WB, SN lysates were performed 20 and 40 min after crush. Samples were collected from a 1 cm piece of SN tissue, holding the compressed area in the medial part, and from contralateral uninjured nerves of the same length. Samples were lysed in lysis buffer (HEPES 10 mmol/L, NaCl 150 mmol/L, SDS 1%, EDTA 4 mmol/L, and protease and phosphatase inhibitors). After centrifugation at 10,000× *g* for 15 min at 4 °C, the supernatants were electrophoresed and processed as previously described [18]. For densitometric quantification with Fiji software, the bands of interest were normalized to the housekeeping protein Calnexin.

### 2.8. Statistical Analysis

Sample size was determined based on data collected in our previously published studies. We used n  =  5 mice/experimental group for electrophysiological analysis. For imaging and cell cultures studies, at least 3 independent replicates were performed. The investigator randomly assigned mice to the various treatment groups. For imaging analysis, the quantitation was conducted by an observer who was blind to the experimental groups. No samples or animals were excluded from the analysis. Data were displayed as histograms with individual values and expressed as means ± sem. GraphPad Prism 10 software was used for statistical analyses. Statistical significance was evaluated using Mann–Whitney test, Kruskal–Wallis test, or the ordinary one-way ANOVA test, depending on experimental conditions. Data were considered statistically different when * *p* < 0.05, ** *p* < 0.01, *** *p* < 0.001, and **** *p* < 0.0001.

## 3. Results

### 3.1. H_2_O_2_ Neutralization Impairs Sciatic Nerve Regeneration and NMJ Recovery of Function Without Affecting Axonal Degeneration

**Figure 1:**
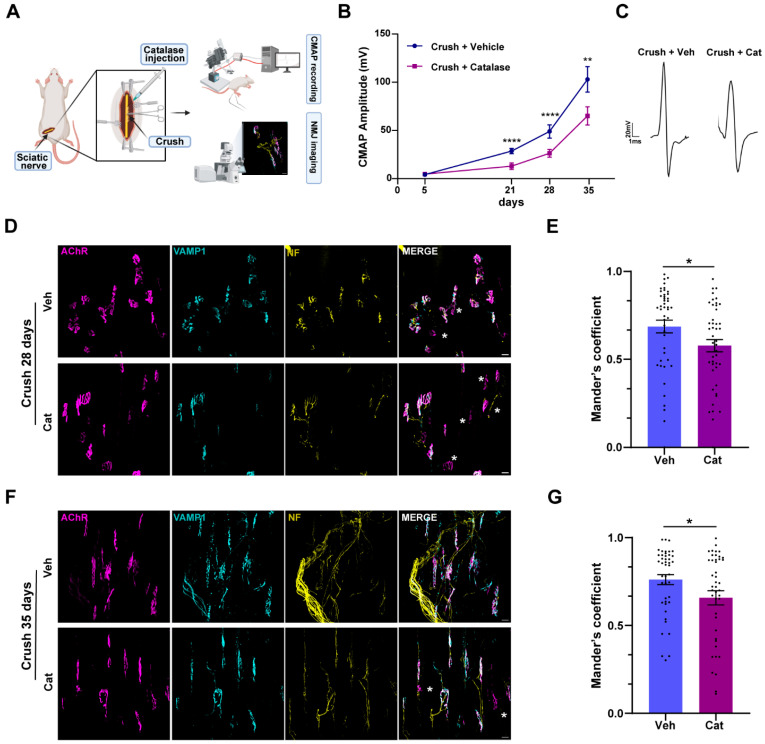
H_2_O_2_ inactivation delayed NMJ functional and structural recovery after sciatic nerve compression. (**A**) Experimental workflow. Created with BioRender.com. (**B**) CMAP recordings were performed on gastrocnemius muscles 5, 21, 28, and 35 days after SN compression, w/wo catalase intra-sciatic administration. Data are expressed as CMAP amplitude (milliVolt, mV). Mixed effect analysis: ** *p* = 0.0011, **** *p* < 0.0001, N = 5. (**C**) Representative CMAP traces 35 days after nerve crush (w/wo catalase). (**D**,**F**) Soleus muscles collected 28 and 35 days post-crush w/wo catalase (Cat) intra-sciatic injection were processed for indirect IF using fluorescent α-BTx to stain post-synaptic AChRs (magenta), anti-VAMP1 (cyan), and anti-neurofilament (NF, yellow) antibodies to identify the pre-synaptic compartment. Asterisks identify degenerated NMJs. Scale bars: 20 µm. (**E**,**G**) Quantification of regenerated NMJs at 28 and 35 days post-crush, respectively, with Mander’s coefficient, which represents the overlap between pre- and post-synaptic markers. Mann–Whitney test: * *p* = 0.0154 (**E**), * *p* = 0.0452 (**G**), N = 3, 15 NMJs analyzed/muscle.

To evaluate the role of H_2_O_2_ in nerve function recovery after injury, we assessed the impact of its neutralization on neurotransmission restoration following a traumatic nerve compression by electrophysiology. We performed an intra-nerve injection of catalase, the enzyme catalyzing the breakdown of H_2_O_2_ into water and oxygen, prior to nerve crush, and monitored functional recovery by measuring the compound muscle action potential (CMAP) in the gastrocnemius muscle 5, 15, 21, 28, and 35 days post-injury, when full recovery was achieved in vehicle-treated animals. Since motor axon degeneration distal to the injury site resulted in neuromuscular junction (NMJ) denervation, we parallelly assessed NMJ integrity through imaging (Figure 1A). Our findings revealed that H_2_O_2_ neutralization significantly delayed neurotransmission recovery, as evidenced by a significant reduction in CMAP amplitudes at all time points analyzed (Figure 1B). Figure 1C shows representative CMAP traces recorded 35 days post-crush, showing that the main peak amplitude was higher in vehicle- vs. catalase-treated samples. Such delay was further confirmed through NMJ immunostaining. We used fluorescently labeled α-bungarotoxin (α-BTX) to visualize post-synaptic acetylcholine receptors (AChRs) and antibodies against VAMP1 and neurofilaments (NFs) to stain the presynapse. The extent of NMJ innervation was quantified using Mander’s coefficient, which measures the colocalization between pre-synaptic VAMP1 and post-synaptic AChR staining. Fully innervated NMJs exhibited coefficient values close to 1, indicating complete overlap of pre-synaptic and post-synaptic structures, whereas denervated endplates had values approaching 0, reflecting the complete degeneration of pre-synaptic nerve terminals. This analysis revealed a significant impairment in NMJ structural recovery at 28 and 35 days post-sciatic-nerve-crush in mice where H_2_O_2_ was inactivated by catalase, compared with controls.

To rule out the possibility that impaired regeneration was due to delayed degeneration caused by catalase, we recorded the CMAP in the gastrocnemius muscle 5 days after SN crush, a time point at which complete nerve degeneration was expected, with or without intra-sciatic injection of catalase [22,23]. As shown by Supplementary Figure S1A, both catalase- and vehicle-treated mice exhibited a comparable deficit in neurotransmission. Consistently, whole-mount sciatic nerve preparations from mice expressing fluorescent ChAT-Tomato-positive axons showed similar axonal damage in the two conditions 20 min after crush, as indicated by the comparable loss of fluorescence signal in the injured area (Figure S1B). This result was further confirmed by quantification of NMJ innervation status using Mander’s coefficient, as previously described, which revealed that the NMJs were structurally intact one day after injury, but were completely denervated by day 5, with no detectable differences between the two groups (Figure S1C–F).

In conclusion, these findings demonstrate that neuronal degeneration following sciatic nerve compression is not affected by H_2_O_2_. Rather, H_2_O_2_ plays a critical role in facilitating the regenerative process.

### 3.2. Injury-Induced H_2_O_2_ Triggers ERK Activation in Schwann Cells

**Figure 2:**
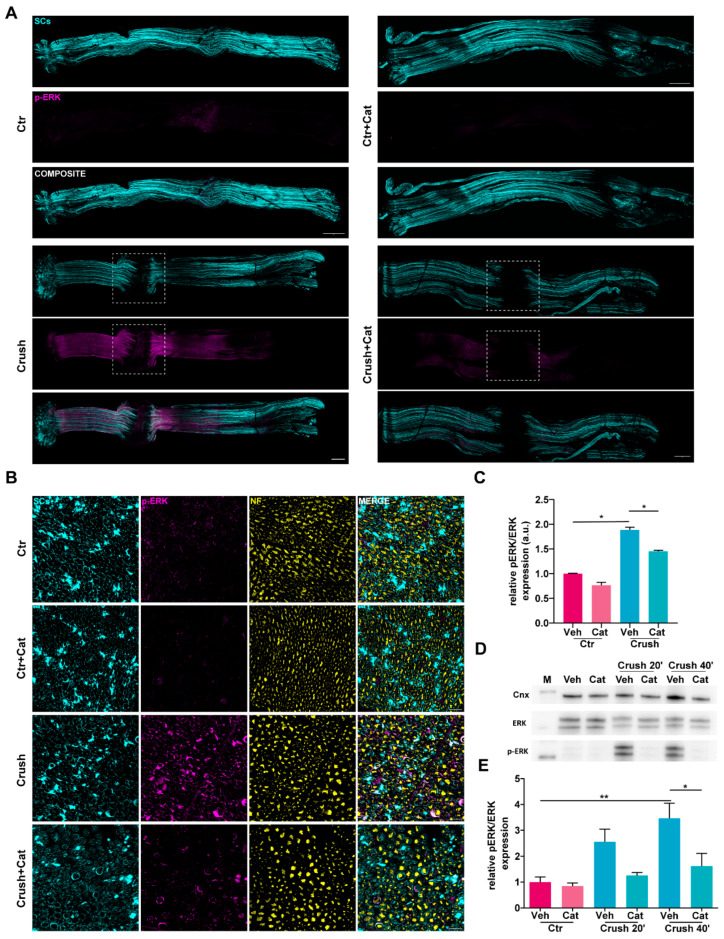
Injury-induced H_2_O_2_ activated ERK signaling in Schwann cells. (**A**) Phospho-ERK 1/2 signal (magenta) in whole-mount SNs 40 min after crush (w/wo catalase intra-sciatic injection). White squares indicate the crush site. SCs (plp-GFP) are in cyan. Scale bars: 500 µm. (**B**) Phospho-ERK 1/2 signal (magenta) in cross sections of SNs 40 min after crush (w/wo catalase). SCs are in cyan, NFs in yellow. Scale bars: 20 µm. (**C**) Quantification of p-ERK signal in cross sections. Mann–Whitney test: * *p* = 0.0286, * *p* = 0.0285. (**D**) WB showing crush-induced ERK1/2 phosphorylation, and its reduction by catalase. Total ERK and Calnexin were used for normalization and as housekeeping for the quantitation in (**E**), respectively. Ordinary one-way ANOVA test: * *p* < 0.05, ** *p* < 0.05.

To investigate whether H_2_O_2_ generated in response to nerve injury influences ERK1/2 signaling in SCs, a key pathway contributing to SC transdifferentiation in response to damage [17], we administered catalase intra-sciatically prior to nerve compression and assessed ERK phosphorylation at 20 and 40 min post-injury, when ERK phosphorylation has been previously observed [24]. Nerve crush induced a robust and rapid activation of ERK1/2 via phosphorylation, as evidenced by increased IF intensity of p-ERK1/2 signal in whole-mount nerve preparations (Figure 2A). Cross-sectional immunofluorescence analysis of SNs showed that the signal was primarily localized within SCs (Figure 2B). In contrast, SNs from mice pretreated with catalase prior to injury exhibited a significant reduction in ERK1/2 phosphorylation compared with those injected with vehicle only (Figure 2C), meaning that ERK phosphorylation was markedly triggered by H_2_O_2_. WB of SN lysates and the relative quantification corroborated the immunofluorescence findings, confirming that ERK1/2 phosphorylation was strongly H_2_O_2_-dependent (Figure 2D,E). Notably, neither compression per se nor treatment with catalase alone altered the total ERK levels (Figure 2D and Figure S2). Together, these findings show that H_2_O_2_ generated after nerve injury promotes the rapid phosphorylation of ERK1/2 without affecting the total ERK content.

### 3.3. H_2_O_2_-Dependent c-Jun UpRegulation in SCs Following Nerve Injury

**Figure 3:**
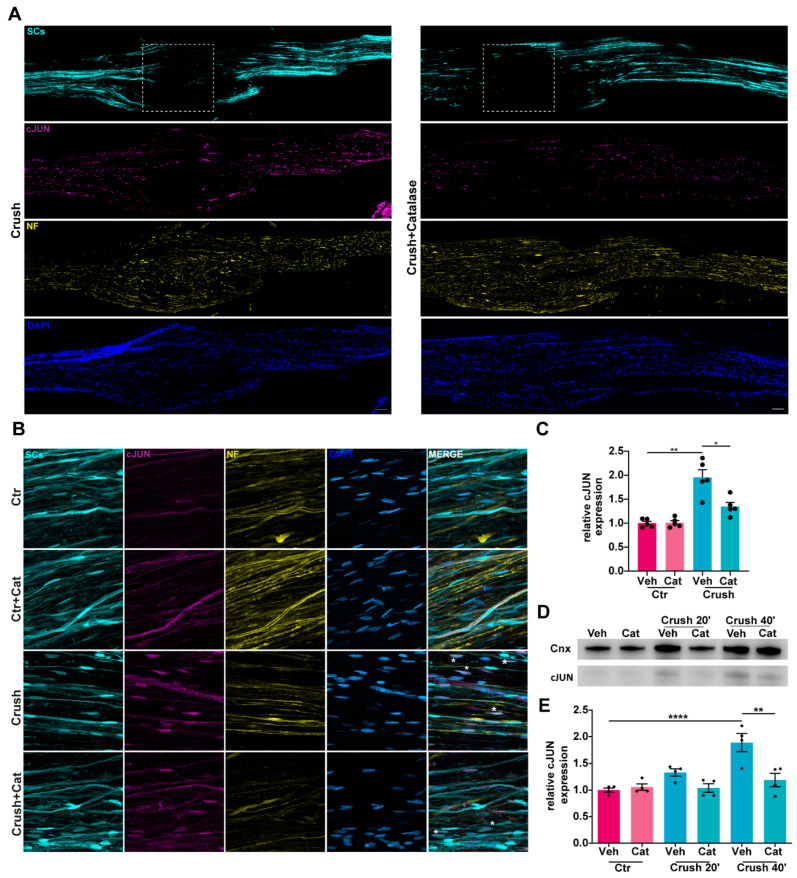
H_2_O_2_-dependent c-Jun upregulation in SCs following nerve injury. (**A**) c-Jun signal (magenta) in whole-mount SNs before and 40 min after crush (w/wo catalase intra-sciatic injection). White squares indicate the crush site. SCs are in cyan, NFs in yellow, and nuclei (DAPI) in blue. Scale bars: 500 µm. (**B**) c-Jun (magenta) in SN longitudinal sections 40 min after crush (w/wo catalase). Asterisks indicate c-Jun-positive SCs. SCs are in cyan, NFs in yellow, and nuclei in blue. Scale bars: 20 µm (**C**) Quantification of c-Jun signal expressed by SCs in longitudinal sections. Mann–Whitney test: * *p* = 0.0159, ** *p* = 0.0079. (**D**) WB showing c-Jun upregulation following crush, which was reduced by catalase. Calnexin was used as housekeeping for the quantitation in (**E**). Ordinary one-way ANOVA test: ** *p* < 0.01, **** *p* < 0.01.

**Figure 4:**
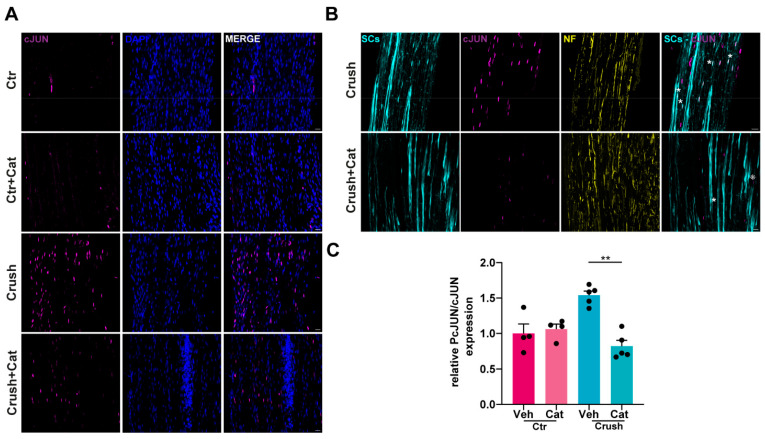
H_2_O_2_-dependent phosphorylation of c-Jun in Schwann cells after nerve injury. (**A**) Phospho-c-Jun signal (magenta) in SN longitudinal sections 40 min after crush (w/wo catalase). Nuclei (DAPI staining) are in blue. Scale bars: 20 µm. (**B**) Phospho-c-Jun detection (magenta) in SN longitudinal sections before and 40 min after crush (w/wo catalase). Asterisks indicate SCs positive for phospho-c-Jun. SCs are in cyan (GFP-positive), NFs in yellow, and nuclei in blue. Scale bars: 20 µm (**C**) Quantification of phospho-c-Jun signal in SCs in longitudinal sections, normalized to total c-Jun. Kruskal–Wallis test: ** *p* = 0.0044.

c-Jun protein levels are rapidly upregulated in SCs in response to injury, where c-Jun functions as a key transcriptional regulator of SC reprogramming into repair SCs [11]. To understand whether injury-induced H_2_O_2_ contributes to c-Jun upregulation, we employed the same experimental approach described above (Figure 1A). Immunofluorescence analysis of whole-mount and longitudinal SN sections revealed a pronounced increase in c-Jun expression in SCs 40 min after crush, which was attenuated in samples where catalase was intra-nerve injected prior to compression (Figure 3A–C). WB analysis, and the correspondent quantitative assessment, showed a H_2_O_2_-dependent c-Jun increase as early as 20 min post-injury (Figure 3D,E). Notably, H_2_O_2_ induced both an upregulation of c-Jun expression in SCs and its phosphorylation, as revealed by immunofluorescence analyses reported in Figure 4A,B and the corresponding quantification (Figure 4C). The same result was obtained upon exposure of primary SCs to H_2_O_2_ (Figure S3). Hence, H_2_O_2_ generated in response to nerve injury promoted the rapid upregulation and phosphorylation of the transcription factor c-Jun within SCs.

## 4. Discussion

The present findings provide compelling evidence that H_2_O_2_ is a key signaling molecule involved in the early response to an acute and mechanical peripheral nerve injury. Indeed, this oxidative signal activates, within minutes after damage, the pro-regenerative extracellular signal-regulated kinase (ERK) pathway, and upregulates and phosphorylates the transcription factor c-Jun, the master regulator of transdifferention that allows myelinating SCs to acquire a repair phenotype [6].

Reactive oxygen species (ROS) are well-known secondary messengers in cellular signaling, playing a critical role in redox biology and immune responses [25,26]. Among ROS, highly unstable and short-lived molecules, such as the superoxide anion, are rapidly neutralized and therefore unlikely to serve as direct signaling mediators. However, superoxide dismutase catalyzes the conversion of superoxide anions into H_2_O_2_, generating a more stable species. H_2_O_2_ rapidly diffuses across cell membranes, a process enhanced by aquaporin water channels [27,28]. This facilitates the transmission of redox signals from the site of production to neighboring cells, where it selectively oxidizes downstream proteins, triggering specific cellular responses. The latter dictate cellular outcomes such as proliferation, survival, regeneration, or cell death, depending on the specific signaling pathways activated [29].

Among its many functions, H_2_O_2_ participates in wound healing and tissue regeneration, enhancing fin and skin innervation in Zebrafish, and tadpole tail regeneration in Xenopus [30,31]. Additionally, H_2_O_2_ oxidative signaling promotes axonal growth and sensory neuron regeneration, highlighting its broader significance in neural repair and tissue restoration [32,33]. In the context of nerve regeneration, H_2_O_2_ likely works at the neuron-glia interface, orchestrating a complex network of interactions that activate specific molecular and genetic programs essential for nerve repair and functional recovery. Indeed, we recently reported that H_2_O_2_ is produced by neuronal mitochondria in response to acute nerve injuries by neurotoxins or anti-ganglioside and complement complexes, an experimental setting mimicking key pathogenetic events of Miller Fisher syndrome, and engaging the ERK1/2 pathway in perisynaptic Schwann cells (PSCs) at the NMJ [9,34]. Additionally, injury-induced H_2_O_2_ promotes directional axonal regrowth toward the original muscle target through the upregulation of the matricellular protein connective tissue growth factor (Ctgf) in SCs [10]. Furthermore, primary SCs exposed to H_2_O_2_ undergo gene expression rewiring, particularly in genes involved in cytoskeletal remodeling and cell migration, and engage local protein synthesis [21]. Collectively, these findings highlight the role of H_2_O_2_-mediated redox signaling as an “immediate injury signal” able to integrate early damage responses with SC activation, in a regenerative perspective.

The present findings confirm the role of injury-induced ROS in regenerative mechanisms, while extending our knowledge of the molecular targets of H_2_O_2_, namely ERK1/2 and c-Jun, two major signaling networks governing the molecular events underpinning injury-associated SC plasticity. ERK1/2 activation following nerve injury facilitates SC de-differentiation and immune cell recruitment, promoting monocyte and macrophage infiltration [12,35]. Concurrently, the rapid upregulation of c-Jun increases the expression of pro-survival and pro-regenerative genes, enhances cytokine production, increases trophic factor expression, and downregulates myelination-associated genes [11,36]. These molecular events initiate key repair mechanisms, including myelin clearance via autophagy, macrophage-mediated debris removal, and the formation of Bungner’s bands, which provide a structural framework for axonal regrowth [37]. Notably, these processes are activated very rapidly, within 30 min to 1 h post-injury [24,38].

To assess the involvement of H_2_O_2_ in this transition, we employed an SN crush injury model, which disrupted axons while preserving SCs and the basal lamina, thus creating favorable conditions for regeneration. Strikingly, in the same experimental setting we recently reported that H_2_O_2_ was produced within the first 5 min following nerve crush, indicating its role as an early mediator in SC-mediated nerve repair [10].

In this study, we found a rapid, H_2_O_2_-dependent activation of the ERK 1/2 pathway, together with the upregulation and phosphorylation of the transcription factor c-Jun in SCs in vivo and in vitro. Notably, c-Jun phosphorylation has been shown to enhance its transcriptional activity and stability [39]. However, in SCs, this phosphorylation appears to be dispensable for c-JUN function, particularly in the context of SC dedifferentiation and myelin clearance [40]. It is important to recognize that the SC injury response at the lesion site differs markedly from that in the distal nerve stump. Since our study focused exclusively on the injury site, future investigation is needed to determine whether similar H_2_O_2_-mediated mechanisms operate within the distal nerve environment. Our study also showed that H_2_O_2_ neutralization not only affects motor axon re-growth but also negatively impacts NMJ recovery of function. In catalase-treated injured mice, the reduced CMAP amplitude and NMJ reinnervation, the latter revealed by a decreased colocalization of pre-synaptic and post-synaptic markers, indicated that H_2_O_2_ is a crucial actor for efficient neurotransmission rescue and effective re-innervation. Importantly, catalase administration does not affect/delay the degenerative events that are initiated by axonal damage [41,42], rather it acts during the regenerative phase by activating SC repair mechanisms, thereby enhancing axonal outgrowth and NMJ reinnervation. These findings are of particular relevance, as inhibition of a single factor is effective in delaying a very complex process such as regeneration. This effect is likely due to the multifaceted role of H_2_O_2_, which influences and regulates multiple interconnected mechanisms involved in tissue regeneration, rather than acting through a singular pathway [43].

Several important questions remain unresolved, particularly regarding the cellular origin of H_2_O_2_ and whether its pro-regenerative effects are mediated exclusively through SCs. Following sciatic nerve compression, multiple cell types, including fibroblasts, resident macrophages, and vascular endothelial cells are affected by the injury. As such, it remains unclear which of these cell populations contribute to H_2_O_2_ production at the injury site. Although it is plausible that multiple cell types are involved, further investigation is needed to clarify their individual contributions. Furthermore, since successful nerve repair depends on coordinated multicellular interactions within the injury microenvironment [5,44,45,46], H_2_O_2_ diffusion from the injury site may directly influence these additional cellular targets, which, in turn, could play a crucial role in propagating or modifying the H_2_O_2_-mediated signaling cascade, further influencing SC behavior. Among these potential targets are inflammatory cells recruited to the injury site, whose chemokine expression can be modulated by H_2_O_2_ [47].

Finally, our analysis was limited to two specific markers of SC activation (ERK1/2 and c-Jun) at very early time points post-injury; however, other intracellular targets, along with epigenetic mechanisms and local protein synthesis pathways, may also be influenced by H_2_O_2_, and could play a key role in nerve regeneration [48,49,50,51,52,53,54]. Moreover, a comprehensive time-course study extending beyond the initial stages of injury, would be critical to determine the temporal dynamics of H_2_O_2_-mediated effects on SC signaling and injury response.

In conclusion, while our study focused on acute mechanical injury, we acknowledge that this model may not fully capture the complexity of chronic or degenerative nerve conditions. Acute injury triggers a rapid, localized, and transient H_2_O_2_ response, in contrast to the sustained, widespread, and potentially harmful oxidative stress associated with chronic or neurodegenerative states. As a result, the role of H_2_O_2_-dependent signaling in SCs may vary considerably across these contexts and warrants further investigation.

## 5. Conclusions

Our study identifies H_2_O_2_ as a localized injury-induced signal that modulates SC c-Jun and ERK1/2 activation and plays a regulatory role in motor axon regeneration.

By linking oxidative signaling to ERK1/2 activation and c-Jun upregulation in SCs, we provide novel insights into how redox biology regulates nerve repair. Future studies should further explore the precise upstream triggers and downstream targets of H_2_O_2_ production and investigate potential interventions that modulate oxidative signaling to improve nerve repair outcomes.

## Data Availability

Data will be available from the corresponding author upon reasonable request.

## Supplementary Materials

The following supporting information can be downloaded at: https://www.mdpi.com/article/10.3390/cells14090671/s1, Figure S1: Catalase-mediated H_2_O_2_ inactivation does not affect the time course of nerve degeneration following sciatic nerve compression; Figure S2: Injury-induced H_2_O_2_ does not affect total ERK levels in SCs; Figure S3: Primary SCs respond to H_2_O_2_ by upregulating c-Jun.
