# Supplementary material for: Hydrogen Peroxide Modulates the Timely Activation of Jun and Erk in Schwann Cells at the Injury Site and Is Required for Motor Axon Regeneration
Source: Cells. 2025 May 3;14(9):671. doi: 10.3390/cells14090671 (PMC12072069; doi:10.3390/cells14090671)
Supplement: Supplementary file 1 [file cells-14-00671-s001.zip › cells-3580290-supplementary.pdf]

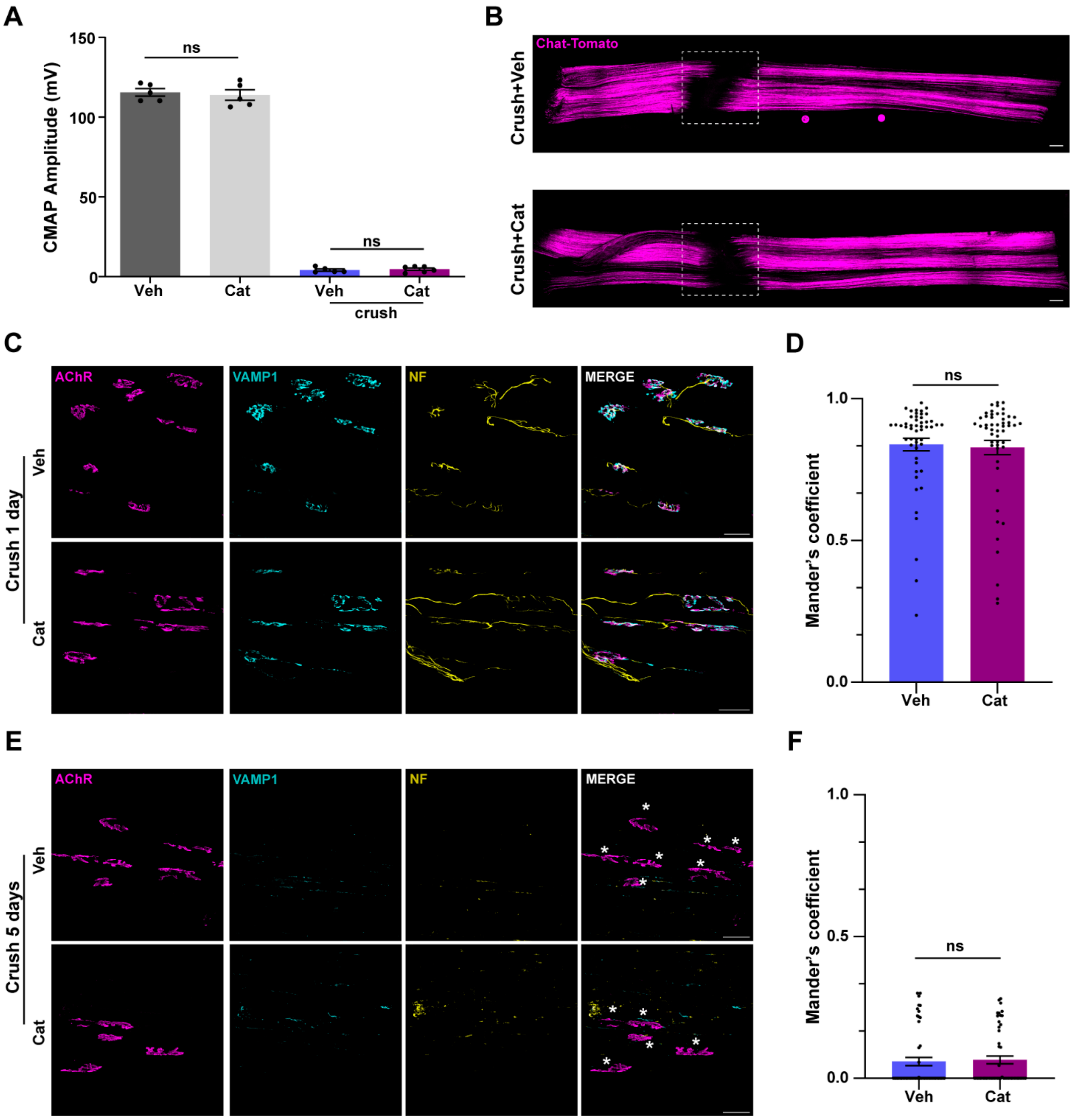

Figure S1. Catalase-mediated H_2O_2 inactivation does not affect the time course of nerve degeneration following sciatic nerve compression

A CMAP recordings performed on gastrocnemius muscles 5 days after SN compression, w/wo catalase intra-sciatic administration. Data are expressed as CMAP amplitude (milliVolt mV). Mixed effect analysis. ns: not significant, N=5. **B** Whole-mount SN immunostaining in mice expressing the fluorescent ChAT-Tomato in motor axons 20 minutes after crush (w/wo catalase). White dotted squares indicate the crush site. Scale bars: 500 μm. **C, D** Soleus muscles collected 1 and 5 days post crush w/wo catalase intra-sciatic administration were processed for indirect immunofluorescence using fluorescent α-BTx to stain post-synaptic AChRs (magenta), and anti-VAMP1 (cyan) and anti-NF (yellow) antibodies to identify the presynaptic compartment. Asterisks identify degenerated NMJs. Scale bars: 20 μm. **D, F** Quantification of regenerated NMJs with Mander's

coefficient that represents the overlap between pre- and post-synaptic markers. Mann Whitney test ns: not significant, n = 3, 15 NMJs analyzed/muscle.

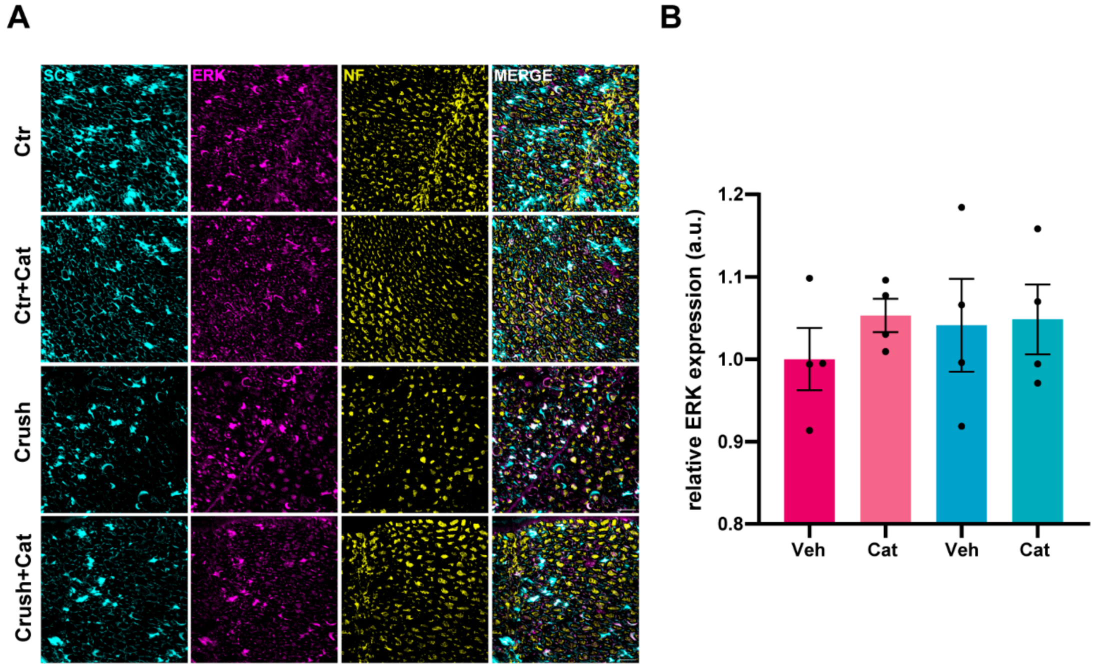

Fig S2 Injury-induced H_2O_2 does not affect total ERK levels in SCs

A ERK signal (magenta) in SN cross sections 40 min after crush (w/wo catalase intra-sciatic injection). SCs are in cyan, NF in yellow. Scale bars: 20 µm. **B** Quantification of total ERK signal in cross sections. Kruskal-Wallis test.

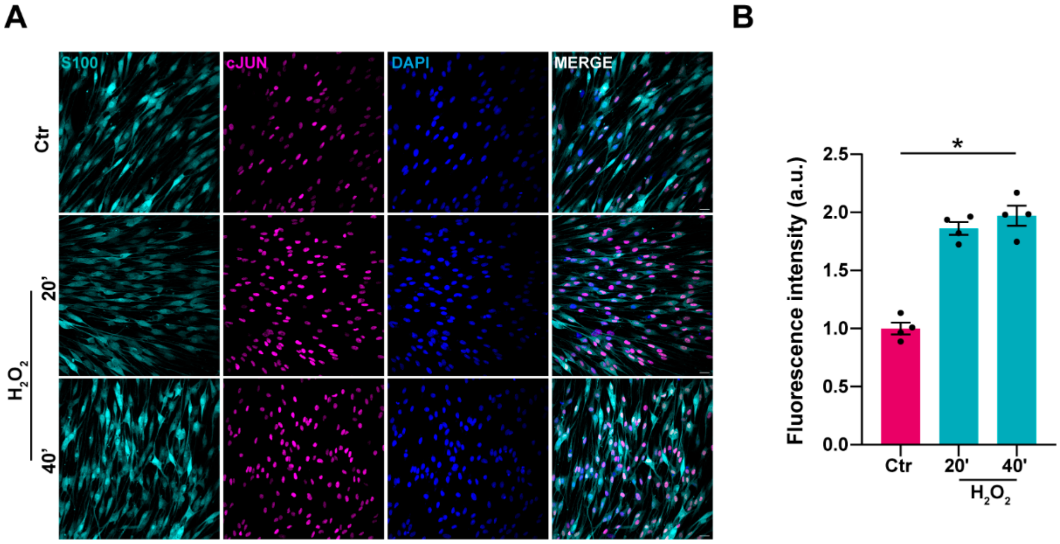

Fig S3. Primary SCs respond to H_2O_2 by upregulating c-Jun

A Time-course of c-Jun upregulation induced in primary SCs by H_2O_2. Immunofluorescence for c-Jun (magenta) in SCs (S100-positive, cyan) after exposure to H_2O_2 (5 µM for 20 or 40 min). Nuclei are stained in blue (DAPI) (Scale bar: 10 µm.) **B** Quantification of c-Jun signal. Kruskal-Wallis test *p =0,0134.
